# Global Profiling in Vestibular Schwannomas Shows Critical Deregulation of MicroRNAs and Upregulation in Those Included in Chromosomal Region 14q32

**DOI:** 10.1371/journal.pone.0065868

**Published:** 2013-06-11

**Authors:** Miguel Torres-Martin, Luis Lassaletta, Jose M. de Campos, Alberto Isla, Javier Gavilan, Giovanny R. Pinto, Rommel R. Burbano, Farida Latif, Barbara Melendez, Javier S. Castresana, Juan A. Rey

**Affiliations:** 1 Neuro-Oncology Laboratory, Research Unit, La Paz University Hospital, IdiPAZ, Madrid, Spain; 2 Department of Otolaryngology, La Paz University Hospital, IdiPAZ, Madrid, Spain; 3 Neurosurgery Department, Fundacion Jimenez Diaz, Madrid, Spain; 4 Neurosurgery Department. La Paz University Hospital, IdiPAZ, Madrid, Spain; 5 Genetics and Molecular Biology Laboratory, Federal University of Piauí, Parnaíba, Brasil; 6 Human Cytogenetics Laboratory, Federal University of Pará, Belém, Brasil; 7 Centre for Rare Diseases and Personalised Medicine, School of Clinical and Experimental Medicine, University of Birmingham, Birmingham, United Kingdom; 8 Molecular Pathology Research Unit, Virgen de la Salud Hospital, Toledo, Spain; 9 Brain Tumor Biology Unit, University of Navarra School of Sciences, Pamplona, Spain; Mayo Clinic, United States of America

## Abstract

**Background:**

Vestibular schwannomas are benign tumors that arise from Schwann cells in the VIII cranial pair and usually present *NF2* gene mutations and/or loss of heterozygosity on chromosome 22q. Deregulation has also been found in several genes, such as ERBB2 and NRG1. MicroRNAs are non-coding RNAs approximately 21 to 23 nucleotides in length that regulate mRNAs, usually by degradation at the post-transcriptional level.

**Methods:**

We used microarray technology to test the deregulation of miRNAs and other non-coding RNAs present in GeneChip miRNA 1.0 (Affymetrix) over 16 vestibular schwannomas and 3 control-nerves, validating 10 of them by qRT-PCR.

**Findings:**

Our results showed the deregulation of 174 miRNAs, including miR-10b, miR-206, miR-183 and miR-204, and the upregulation of miR-431, miR-221, miR-21 and miR-720, among others. The results also showed an aberrant expression of other non-coding RNAs. We also found a general upregulation of the miRNA cluster located at chromosome 14q32.

**Conclusion:**

Our results suggest that several miRNAs are involved in tumor formation and/or maintenance and that global upregulation of the 14q32 chromosomal site contains miRNAs that may represent a therapeutic target for this neoplasm.

## Introduction

Schwannomas are benign tumors that arise from Schwann cells in the peripheral nerves. These tumors often originate from the vestibular nerve, and although they are histologically benign, vestibular schwannomas may cause hearing loss, tinnitus, facial palsy, and when large enough, brain stem compression and even death. Vestibular schwannomas may appear unilaterally but may also appear bilaterally when associated with neurofibromatosis type 2 syndrome (NF2). Patients with NF2 also develop other tumors such as meningiomas and gliomas [1]. The molecular hallmark of the disease is the biallelic inactivation of the tumor suppressor *NF2* gene by several mechanisms [2], such as mutation or loss of heterozygosity (LOH) of chromosome 22 where this gene is hosted (i.e., 22q12.2). Since the first description of monosomy 22 in schwannomas by cytogenetic analyses [3], other genetic alterations in these tumors have been identified, including chromosomal gains of 9q34 and 17q and losses of 1p [4]–[6]. For an extensive review, see citation [7]. Contrary to non-head and neck schwannomas, sporadic vestibular schwannomas have shown no mutations on *BRAF*, *EGFR*, *PIK3CA* or *KRAS* [8]. Controversial findings on epigenetic aberrant methylation of *NF2* in schwannomas have been provided [9]–[12], and data on promoter methylation of tumor-related genes have also been described [13]. The *NF2* gene encodes for Merlin or Schwannomin [14], [15], a protein that shares sequence homology with members of the ezrin/radixin/moesin (ERM) family. Merlin is involved in an array of signaling pathways, such as the suppression of tumorigenesis by its entering into the nucleus and binding to DCAF1 through the blocking of CRL4^DCAF1^ action [16], or downregulation of membrane levels of ErbB2, ErbB3 and EGFR upon cell-to-cell contact [17], [18]. Using microarray technology in schwannomas, complete genome expression analysis is possible, and several regulatory pathways and specific genes, such as *CAV1,* have been found to display an altered expression pattern [19]–[23]. MicroRNAs (miRNAs) are endogenous, non-coding RNAs 21 to 23 nucleotides in length that regulate gene expression at the post-transcriptional level by, among other mechanisms, binding and repressing target mRNAs [24]. Each miRNA is able to regulate thousands of mRNAs, and each miRNA acts in a tissue-specific manner [25]. Overexpression of miRNAs may involve tumor development by silencing certain tumor suppressor genes. On the other hand, underexpression of miRNAs may cause tumor progression by upregulating target oncogenes. Thus, miRNAs might be considered oncogenes or tumor suppressor genes in and of themselves. A wide variety of tumors have been found with altered miRNA expression, such as breast cancers [26], hepatocellular carcinomas [27], osteosarcomas [28] and many others [29]. Aberrant expression patterns in miRNAs have also been described in tumors that affect the nervous system, such as gliomas and meningiomas [30], [31].

In schwannomas, the regulation of two miRNAs has been associated with tumorigenesis: miR-21 upregulation [32] and miR-7 downregulation [33], which suggests that deregulation of these small RNAs plays a role in the development of these tumors. In the light of these results, we performed a microarray analysis on 16 schwannomas and 3 control-nerves for non-coding RNAs, in order to identify new targets for the disease.

## Materials and Methods

### Patient Samples

The study group consisted of 16 patients who underwent surgery at our center for the removal of vestibular Schwannoma. Fifteen of these patients presented sporadic schwannomas and 1 sample (tumor S1) was from a patient diagnosed with NF2. The population included 7 women and 9 men. The mean age of the patients was 45.2±14.9 years. The local ethics review board of the University Hospital La Paz approved the study protocol, which was based on the principles of the Declaration of Helsinki. All patients received detailed information on the study and provided their written informed consent prior to their inclusion in the study.

### DNA/RNA Preparation

DNA was isolated using the Wizard Genomic DNA Purification Kit (Promega). DNA was also extracted from the corresponding patients' peripheral blood. Total RNA was extracted using the mirVana miRNA Isolation Kit (Ambion, CA, USA). A hypoglossal ansa cervicalis nerve and two auricular nerves from non-tumoral patients were used as control samples.

### Mutational and MLPA Analysis of *NF2* and LOH of Chromosome 22q

We performed a mutational analysis of the *NF2* gene by dHPLC/PCR and a specific *NF2* SALSA of MLPA (MRC-Holland) and tested the LOH status of chromosome 22q. In brief, the LOH of chromosome 22q was tested using 5 microsatellite markers (D22S275, D22S264, D22S929, D22S268 and D22S280 located at 22q11-q12.3). For the PCR/dHPLC analysis of the *NF2* gene, a set of 15 primer pairs were selected using the standard PCR method and the dHPLC manufacturer's protocols. For the MLPA analysis, SALSA P044, which contains probes for all *NF2* exons, was used. Complete procedures were described previously [34].

### miRNA Expression Array

We used the GeneChip miRNA 1.0 array (Affymetrix) with coverage of miRBase v.11. The array contains 4 [35] probe sets for mature miRNA and is able to detect the expression of 847 human mature miRNAs and 922 small nucleolar RNAs (snoRNAs) and small Cajal body-specific RNAs (scaRNAs). Hybridization targets were prepared from 500 ng of total RNA using the FlashTag Biotin HSR Kit (Genisphere). The labeled samples were hybridized to GeneChip miRNA arrays (Affymetrix). GeneChips were scanned in a GeneChip Scanner 3000 (Affymetrix). CEL files were generated from DAT files using AGCC software (Affymetrix). To generate the log2 expression estimates, the overall array intensity was normalized between the arrays, and the probe intensity for all probes in a probeset were summarized to a single value using the RMA (Robust Multichip Average) algorithm [36]. The arrays were processed at the IRB Barcelona Functional Genomics Core Facility. The microarray data was entered into the NCBI Gene Expression Omnibus database and are accessible through GEO Series accession number GSE43571.

### Statistical Array Analysis

Given that the 19 samples were processed in two separate batches, with controls and tumors in both, the batch-removal tool of the Partek Genomic Suite 6.6 was used to remove the batch effect. We also used this software in order to include genes as deregulated, and we selected those genes with at least a 2-fold change in expression and a p<0.05 cutoff in the one-way ANOVA test. The rest of statistical analyses were performed using MultiExperiment Viewer (MeV) [37]. Principal component analysis (PCA) was performed by eigenvalue decomposition of the 3 principal components for three-dimensional classification of the samples, and an unsupervised hierarchical cluster by Pearson’s correlation was selected to group the samples and the deregulated miRNAs. We used the mirWalk (http://www.umm.uni-heidelberg.de/apps/zmf/mirwalk/index.html) and DAVID (http://david.abcc.ncifcrf.gov/) web tools in order to predict the biological meaning of the deregulated miRNAs [38], [39]. As NF2 vestibular schwannoma had the same way as the other tumors, we decided to include it in the statistical study.

### Validation by qRT-PCR

For validation by miRCURY locked nucleic acid (LNA)™ Universal RT microRNA PCR protocol qRT-PCR (Exiqon), we used the 3 control nerves and 7 of the 16 Schwannoma chosen at random. A total of 10 miRNAs with aberrant expression were selected based on potential pathways of interest and/or chromosomal location (hsa-miR-1, hsa-miR-10b, hsa-miR-133b, hsa-miR-183, hsa-miR-206, hsa-miR-221, hsa-miR-370, hsa-miR-431, hsa-miR-493 and hsa-miR-720). The cDNA synthesis and real-time amplification were performed as recommended by the manufacturer. All tests were performed in duplicate.

The calculation of gene expression was conducted as follows: Average cycling threshold (Ct) values were obtained using SDS 2.2 software (Applied Biosystems). The maximum Ct value was set at 40. Ct values were normalized using two housekeeping non-coding RNAs extracted from data obtained in arrays (SNORD49A and hsa-miR-130). The relative expression level of each target gene was expressed as ΔCt = C_tref_–Ct_gene_ [40]. Reference-normalized expression measurements were adjusted by defining the lowest expression value as 0, with subsequent 1-unit increases reflecting an approximate doubling of the RNA. The non-parametric Mann-Whitney-Wilcoxon test at p<0.05 coupled with the Benjamini-Hochberg correction test was used to calculate the significance of differences between the control samples and the schwannomas.

## Results

### Allelic Status of 22q and Mutational Analysis of *NF2*

A total of 9 samples (56%) presented 22q LOH using microsatellite markers. Eight of the schwannomas (50%) had NF2 sequence mutations detected by PCR/dHPLC, and 10 of the schwannomas (62%) had NF2 sequence mutations detected by the combined MLPA and PCR/dHPLC analysis. In 7 cases, LOH was concomitant with either MLPA or PCR/dHPLC alteration. In 4 cases, no alterations were found. We found no mutations in the patients’ peripheral blood, with the exception of the NF2 patient. The individual results are shown in Table S1.

### Deregulation in miRNAs

**Figure 1:**
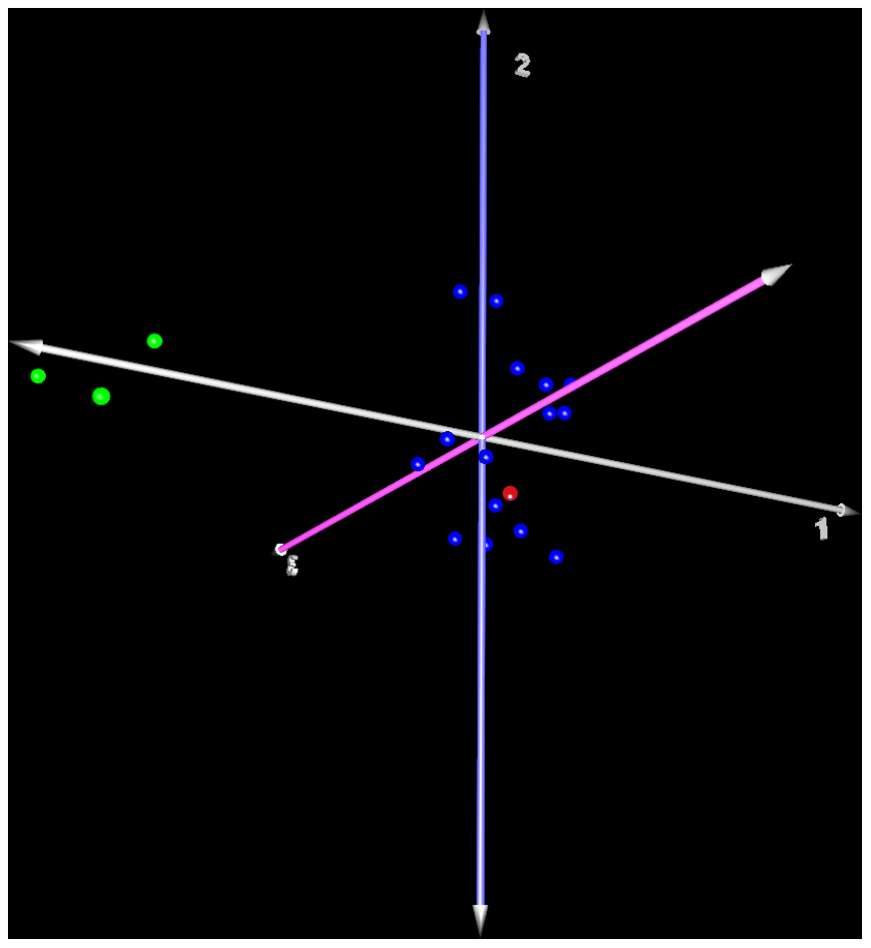
Three dimensional representation of the Principal Component Analysis of the 16 schwannomas (blue dots corresponding to sporadical and red dot to the NF2 patient) and the 3 control nerves (green dots).

**Figure 2:**
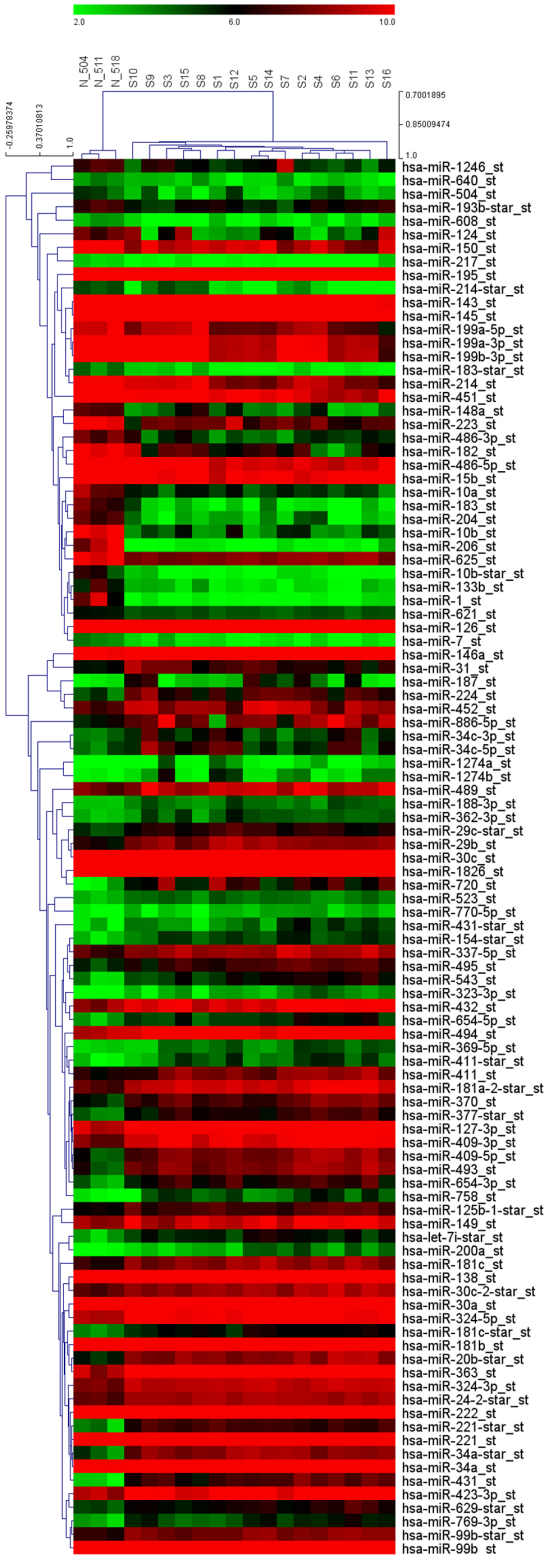
Hierarchical cluster by Pearson’s correlation of all 19 samples (16 schwannomas and 3 control nerves) and miRNAs matching the criteria established as deregulated (2-fold change in expression and a p<0.05 cutoff by one-way ANOVA).

When the control nerves and the schwannomas were compared using the one-way ANOVA at p<0.05 and with at least a 2-fold change, we found 176 deregulated miRNAs from a total of 847 human miRNAs available in the array. Of these, 119 (14%) were found to be upregulated and 57 (6.7%) were downregulated. The top 30 of each group are shown in Tables 1 and 2, and the full results of the deregulation can be seen in Table S2. The PCA showed a clear distinction between the control nerves and the schwannomas, and the tumor from the NF2 patient grouped together with the other schwannomas (Figure 1). The hierarchical cluster of deregulated genes showed no major differences at the miRNA level between the schwannomas (included that tumor from a NF2 patient) but showed differences with respect to the controls (Figure 2).

**Table 1:** The top 30 upregulated miRNAs obtained by microarray analysis, ordered by positive fold-change in schwannomas.

miRNA	p-value	Fold-Change
hsa-miR-431	5.47E-09	29.93
hsa-miR-720	4.96E-05	16.40
hsa-miR-34a*	2.73E-09	14.80
hsa-miR-221*	1.04E-09	9.96
hsa-miR-21	0.00116	9.71
hsa-miR-493	4.23E-07	8.48
hsa-miR-409-5p	9.25E-07	8.31
hsa-miR-363*	0.00010	8.09
hsa-miR-363	4.86E-09	8.05
hsa-miR-154	2.85E-05	7.53
hsa-miR-654-3p	0.00001	7.45
hsa-miR-20b*	3.33E-08	7.14
hsa-miR-543	3.29E-05	6.79
hsa-miR-377*	5.19E-06	5.63
hsa-miR-758	0.00057	5.63
hsa-miR-22*	5.70E-05	5.51
hsa-miR-127-3p	9.35E-07	5.42
hsa-miR-542-5p	0.00140	5.34
hsa-miR-200a	0.00028	5.31
hsa-miR-34a	4.27E-13	5.23
hsa-miR-224	0.00052	5.23
hsa-let-7i*	5.30E-06	5.17
hsa-miR-409-3p	9.77E-06	4.98
hsa-miR-221	1.23E-12	4.82
hsa-miR-184	0.01893	4.73
hsa-miR-181a-2*	2.89E-06	4.65
hsa-miR-493*	0.00094	4.65
hsa-miR-410	0.00096	4.47
hsa-miR-222	1.28E-10	4.47
hsa-miR-181c*	1.35E-07	4.30

**Table 2:** The top 30 downregulated miRNAs obtained by microarray analysis, ordered by negative fold-change in schwannomas.

miRNA	p-value	Fold-Change
hsa-miR-206	2.17E-09	**−**222.68
hsa-miR-1	3.85E-09	**−**62.96
hsa-miR-10b	4.85E-08	**−**51.58
hsa-miR-183	1.01E-06	**−**25.33
hsa-miR-182	0.00019	**−**24.44
hsa-miR-204	3.57E-06	**−**17.68
hsa-miR-10b*	5.15E-08	**−**14.51
hsa-miR-133b	8.36E-08	**−**11.44
hsa-miR-214	2.59E-05	**−**11.13
hsa-miR-383	7.99E-06	**−**10.03
hsa-miR-223	0.000085	**−**7.73
hsa-miR-148a	0.000486	**−**7.61
hsa-miR-34b	0.005987	**−**7.36
hsa-miR-10a	0.000004	**−**7.31
hsa-miR-486-3p	0.000185	**−**6.58
hsa-miR-509-3p	1.14E-05	**−**6.57
hsa-miR-486-5p	9.88E-05	**−**6.27
hsa-miR-199a-3p	0.002156	**−**5.42
hsa-miR-199b-3p	0.002919	**−**5.33
hsa-miR-133a	4.49E-06	**−**5.18
hsa-miR-214*	0.015433	**−**4.01
hsa-miR-15b	2.10E-05	**−**3.87
hsa-miR-625	3.35E-07	**−**3.76
hsa-miR-143	0.000495	**−**3.74
hsa-miR-138-2*	0.000303	**−**3.59
hsa-miR-183*	2.30E-05	**−**3.55
hsa-miR-143*	0.01991	**−**3.29
hsa-miR-92a-1*	0.01092	**−**3.23
hsa-miR-595	8.73E-05	**−**3.17
hsa-miR-199a-5p	0.02445	**−**3.10

The chromosomal location of the miRNA affected by deregulation is shown in Table 3. Chromosome 14 was the most affected, with 52% of all miRNAs located on this chromosome deregulated; all of these miRNAs were overexpressed, but one was downregulated. Chromosome X also presented a high degree of deregulation, with more than 27% of all miRNAs in this chromosome showing aberrant expression. Other chromosomes displaying significant rates of abnormally expressed miRNAs were chromosome 1 (28%), chromosome 7 (26%) and, although fewer miRNA probes were available in the array for this study, chromosome 6 (29%) and 18 (33%).

**Table 3:** Available miRNAs correspond to those miRNAs tested in the microarray.

Chromosome	Available miRNAs	Up-regulated	Down-regulated	Total	Percentage of deregulation (%)
1	61	14	3	17	27.9
2	23	2	3	5	21.7
3	35	2	2	4	11.4
4	31	0	2	2	6.5
5	30	2	4	6	20.0
6	17	3	2	5	29.4
7	42	4	7	11	26.2
8	27	1	3	4	14.8
9	30	1	4	5	16.7
10	23	1	2	3	13.0
11	38	3	2	5	13.2
12	42	2	0	2	4.8
13	25	0	2	2	8.0
14	81	40	2	42	51.9
15	27	4	1	5	18.5
16	17	4	2	6	35.3
17	48	6	4	10	20.8
18	9	0	3	3	33.3
19	102	9	4	13	12.7
20	19	0	0	0	0.0
21	7	1	0	1	14.3
22	19	0	1	1	5.3
X	89	20	4	24	27.0

Up or downregulation was obtained from those with at least a 2-fold change and p>0.05 when schwannomas and control nerves were compared. Other non-coding RNAs were not considered in this table.

### Deregulation in other Non-coding RNAs

Other non-coding RNAs appeared deregulated: 128 of all snoRNAs available in the array corresponded to HAC-Box, with one downregulated (U71c) and 25 upregulated. A total of 67 of the 274 CD-box snoRNAs were upregulated, and only 5 of the other 499 snoRNAs were upregulated. The scaRNAs were not deregulated, and the 5.8s rRNAs were upregulated in all 10 copies available at the probeset. The 30 most deregulated non-coding RNAs are shown in Table 4.

**Table 4:** The top 30 deregulated non-miRNAs present in the arrays.

Transcript ID	Chr	Sequence	p-value	Fold-Change
HBII-99	20	CDBox	1.14E-07	5.97
14q(II-12)	14	CDBox	6.83E-07	5.41
U48	6	CDBox	7.30E-06	5.05
14q(I-4)	14	CDBox	8.33E-06	5.05
U107	X	HAcaBox	2.61E-09	4.95
HBII-180C	19	CDBox	7.09E-09	4.90
14q(II-14)	14	CDBox	2.52E-05	4.85
U43	22	CDBox	3.53E-07	4.46
U31	11	CDBox	3.73E-06	4.42
14q(II-3)	14	CDBox	8.57E-06	4.42
U43	22	CDBox	7.81E-07	4.31
ENSG00000200879	11	snoRNA	7.21E-11	4.24
14q(II-14)	14	CDBox	5.42E-05	4.20
ACA7	3	HAcaBox	3.00E-06	4.09
U46	1	CDBox	7.68E-08	4.06
U8	17	CDBox	1.82E-06	4.02
ACA48	17	HAcaBox	6.70E-07	3.98
ENSG00000202252	11	snoRNA	3.03E-05	3.91
U8	17	CDBox	3.32E-07	3.87
U46	1	CDBox	4.34E-07	3.78
14q(II-26)	14	CDBox	0.00128	3.76
ACA16	1	HAcaBox	7.13E-05	3.70
ENSG00000207118	11	snoRNA	0.00011	3.68
U103	1	CDBox	3.05E-08	3.68
ACA48	17	HAcaBox	1.64E-06	3.61
ACA16	1	HAcaBox	3.75E-06	3.54
HBII-85-8	15	CDBox	3.89E-07	3.49
U33	19	CDBox	4.22E-08	3.44
14q(II-12)	14	CDBox	3.84E-05	3.43
HBII-180A	19	CDBox	5.51E-08	3.43

### Web-tool Analysis

Using mirWalk, a web-tool that finds gene targets from a given list of miRNAs, we obtained a set of validated gene targets using our lists of upregulated and downregulated miRNAs. These lists of targets, with 1554 and 1597 genes respectively, were analyzed separately using the DAVID platform, which uses a set of functional annotation tools to find biological meaning from data using gene ontology terms, BioCarta, etc. Using these web tools, the main deregulated pathways identified were vasculature and nervous system development (Tables S3 and S4).

### Microarray Data Correlation versus *NF2* Alterations and 22q Allelic Status

We also tested the possibility that grouping schwannomas by their molecular characteristics such as the presence or not of *NF2* mutations or LOH 22q/normal 22q, would result in a statistically significant subset of over- or under-expressed miRNAs (using the one-way ANOVA at p<0.05 and at least a 2-fold change). Fourteen miRNAs matched these criteria, and a number of the miRNAs displayed statistical significance in several comparisons (Table S5). These included the presence of 22q LOH vs. normal constitution (with significant upregulation of miR-195* in miRNAs with LOH), the presence of sequence alteration by all methods vs. normal miRNAs (4 miRNAs), sequence alteration exclusively by PCR/dHPLC vs. normal miRNAs (3 miRNAs), and one or more detected alterations vs. no detected alterations (9 miRNAs).

### Validation by qRT-PCR

Validation of the expression patterns of 11 miRNAs and SNORD49A obtained through microarray analysis by performing qRT-PCR (Table 5). In all cases, the trend observed in the microarrays (upregulation, downregulation or no deregulation) was confirmed by this technique.

**Table 5:** The qRT-PCR showed all miRNAs significantly up or downregulated in schwannomas compared to control-nerves (at least a 2-fold change and p>0.05), except in those two used as house-keeping (SNORD49A and miR-103).

miRNA	Location	Fold-change	P-value
miR-1	20q13.33	126.72	0.0251
miR-10b	2q31.1	269.19	0.0251
miR-133b	6p12.2	210.49	0.0251
miR-183	7q32.2	57.34	0.0251
miR-206	6p12.2	379.28	0.0251
miR-221	Xp11.3	–9.62	0.0251
miR-370	14q32.2	–3.13	0.0364
miR-431	14q32.2	-6.38	0.0251
miR-493	14q32.2	–7.99	0.0251
miR-720	3q26.1	–3.49	0.0364
SNORD49A	17p11.2	1.01	0.7324
miR-103	5q34	–1.01	0.7324

## Discussion

In this study, we used 16 schwannomas and 3 control nerves to analyze the expression levels of miRNAs and other non-coding RNAs in Affymetrix microarrays. As an alternative for control purposes, in vitro cultures of Schwann cells may be used. However, although it is possible to obtain up to 90% purity of Schwann cells [41], it has been reported that Schwann cells in cultures modify their native gene expression [42]. Thus, it appears that peripheral nerve may be a more reliable control in expression studies, as it has been proved by several reports [20], [21], [33]. Our findings showed deregulation in schwannomas of more than 150 miRNAs and a set of other non-coding miRNAs, with special upregulation of those miRNAs located in the chromosomal 14q region. Eleven of the miRNAs and 1 snoRNA were validated by qRT-PCR.

The most downregulated miRNAs in this study include the myomiRs, composed of three clusters: miR-1-1/miR-133a-2, miR-1-2/miR-133a-1, and miR-206/miR-133b. These miRNAs have been identified as typically downregulated in several types of cancer, such as colorectal cancer and hepatocellular carcinoma (reviewed in citation [43]). Other miRNAs with reduced expression in our series included miR-10b, which has also been associated with other cancers such as gliomas [44], metastatic breast carcinomas [45] and pancreatic carcinomas [46]; however, in all of these cases, miR-10b was upregulated [44]. Decreased expression of this miRNA has been reported to escape senescence by Argonaute 2 expression in stem cells [47]. The miRNA 183/182 cluster, which was downregulated in our series, has also been found to be deregulated in medulloblastoma [48] and prostate cancer [49], and the cluster has been shown to inhibit cell proliferation and migration by targeting *FGF9* and *NTM* in Schwann cells [50]. Furthermore, miR-183 has been found to be capable of regulating Ezrin protein (a member of the family with similarity to Merlin) in osteosarcoma and lung cancer [51], [52]. The miRNA 183/182 cluster may therefore be involved in Schwannoma formation if it plays a similar role in these neoplasms. In malignant peripheral nerve sheath tumors (MPNSTs), miR-204 is downregulated [53], in a similar pattern as that found in the schwannomas in our arrays. As these tumors also developed from Schwann cells, there may be common features associated with this particular miRNA that appear to participate in the development of both neoplasms.

The upregulated miRNAs in our studies include miR-21, which targets PTEN in non-small cell lung cancer [54]; cluster miR-221/miR-222, which is activated in Schwann cell proliferation following sciatic nerve injury by targeting *LASS2* [55] and accelerates proliferation during liver regeneration [56]; and miR-370, which is capable of reducing *NF1* mRNA levels in acute myeloid leukemia [57] and is also overexpressed in prostate cancer [58]. Another miRNA with increased expression is miR-493, which is defined as a tumor suppressor in bladder cancer and is capable of downregulating FZD4 and RhoC protein expression [59]. Thus, several of these miRNAs may contribute to the benign proliferation observed in schwannomas, such as miR-221/miR-222, due to their demonstrated capacity to affect proliferation and regeneration in various tissues, including nerve tissue.

Web-tool analysis (performed using DAVID) provides us with the theoretical results of validated genes affected by miRNA deregulation (performed with mirWalk) and the pathways and related components that involve these genes. The DAVID results showed several processes related to carcinogenesis in the Kyoto Encyclopedia of Genes and Genomes (KEGG), including hsa05215:Prostate cancer, hsa05214:Glioma and the hsa04012:ErbB signaling pathway. However, when the up- or down-regulated miRNAs we had previously identified were considered, the findings showed similar deregulated GO-terms (e.g., GO:0010604 positive regulation of the macromolecule metabolic process; GO:0051960 regulation of nervous system development; GO:0051270 regulation of cell motion, etc.). Therefore, the data obtained from the analysis of our deregulated miRNAs with these web-tools must be viewed with caution, and no definitive conclusions should be drawn. This event might be related to the lack of tissue specificity when selecting deregulated miRNAs that were validated for target genes. Once more detailed data are available on this subject, a re-evaluation of the pathways affected by miRNA interaction may be performed. We previously analyzed the critical regulatory pathways that are abnormally expressed in schwannomas by studying the expression levels of 96 tumor-related genes [21]. Those genes coding for proteins related to apoptosis and angiogenesis and genes related to DNA damage repair were the most frequently altered.

When the chromosomal location of the abnormally expressed miRNAs was analyzed, we found a clear upregulation for those miRNAs located in the chromosomal 14q32 region. Only two miRNAs, miR-203 and miR-625, had reduced expression, while approximately 40 miRNAs showed overexpression, including miR-431, miR-370 and miR-493, as validated by qRT-PCR. This region involves a large miRNA cluster [60] [61] altered in several neoplasms such as gliomas (by downregulation) [62] and a subtype of acute myeloid leukemia (by upregulation) [63]. Consequently, this region also appears to play a pivotal role in Schwannoma formation and/or maintenance. On chromosome 22, which usually suffers a loss of heterozygosity in Schwannoma, 5% of the miRNAs located in this region appeared deregulated. In fact, only miR-185 (at 22q11) displayed reduced expression levels. This finding suggests that, in schwannomas, losses involving 22q do not seem to affect the expression pattern of miRNA located there. However this particular miRNA (miR-185) may be affected as a result of other yet unknown regulatory molecular mechanisms.

Several deregulated miRNAs we identified concur with the data from previous reports in schwannomas [32], [33]. In agreement with the data, miR-7, miR-638, miR-143* and miR-498 were downregulated, and miR-221, miR-21, miR-29, miR-30a and miR-138 were upregulated in our study. On the other hand, a few of the miRNAs, such as miR-34a, let-7d and miR-451, did not match the same trend of aberrant expression as that previously described. Establishing a reason for these divergences is a difficult task, because neither the number of samples nor the methodology used should influence the result. Therefore, our findings generally concur with the results of previous reports, although several miRNAs with a different expression pattern were identified.

We found alterations in other non-coding RNAs (snoRNAs and scaRNAs) using this type of array. These alterations have been associated with the development or progression of breast cancer and acute leukemia [64], [65]. Our results showed that at least 98 of these non-coding RNAs were deregulated, almost all by increased expression, except for HACA-Box SNORA71C. A total of 14 SnoRNAs were also located in chromosome 14q32, highlighting the paramount role played by the miRNAs located in this genomic region in Schwannoma development.

Schwannomas with specific molecular characteristics, such as gene mutations of *NF2* and/or LOH of 22q region, may present a different miRNA expression pattern when compared to unaltered schwannomas. When comparing levels of miRNA expression obtained from arrays with other molecular studies performed on these tumors (*NF2* mutation detection or 22q allelic status determination), we found 14 miRNAs with aberrant expression. For example, abnormal overexpression of miR-155, which is associated with schwannomas with no alterations of any sort, displayed a statistical significance of p<0.001; however, miR-125b-2* was found to be significantly downregulated in those tumors carrying a mutation (p<0.01). Despite these differences, establishing a molecular reason for these particular alterations is difficult. Furthermore, the p-values were relatively high compared with those obtained when tumors and controls were studied, and outliers could not be ruled out as playing a role in these results. We therefore concluded that, at the miRNA level, a few miRNA subsets may have relevance depending on the genetic status of the tumor (the presence of *NF2* mutation and/or LOH of 22q), although more research needs to be conducted on this particular aspect.

In conclusion, our results show that the non-coding RNA expression pattern is critically affected in schwannomas when compared to healthy tissue. Of these, miRNAs seems to be the most frequently affected, given that at least 20% appear deregulated, including several depicted in other tumors. These mRNAs include miR-10b and miR-204. Furthermore, a well-described cluster of non-coding RNAs in the chromosomal region 14q32 seems to present global upregulation, suggesting that miRNA deregulation in this region might play a significant role in the development and/or maintenance of these tumors. Finally, other non-coding RNAs, although less numerous when compared with miRNAs, also seem to be altered in schwannomas.

## Supporting Information

Table S1 **Mutations detected on vestibular schwannomas and LOH status of chromosome 22q.**
(XLSX)

Table S2 **Deregulation of all human probes in microarrays.** Output obtained from Partek Genomic Suite 6.6 for one-way ANOVA.
(XLSX)

Table S3 **Downregulated pathways obtained by DAVID.**
(XLSX)

Table S4 **Upregulated pathways obtained by DAVID.**
(XLSX)

Table S5 **Deregulation profile in miRNA based on molecular characteristics of schwannomas.**
(XLSX)
